# Effects of Janus Kinase Inhibitors on Rheumatoid Arthritis Pain: Clinical Evidence and Mechanistic Pathways

**DOI:** 10.3390/biomedicines13102429

**Published:** 2025-10-05

**Authors:** Andrej Belančić, Seher Sener, Yusuf Ziya Sener, Almir Fajkić, Marijana Vučković, Antonio Markotić, Mirjana Stanić Benić, Ines Potočnjak, Marija Rogoznica Pavlović, Josipa Radić, Mislav Radić

**Affiliations:** 1 Department of Basic and Clinical Pharmacology and Toxicology, Faculty of Medicine, University of Rijeka, Braće Branchetta 20, 51000 Rijeka, Croatia; 2 Department of Pediatric Rheumatology, Adana City Training and Research Hospital, Adana 01370, Türkiye; kzl_seher@hotmail.com; 3 Department of Cardiology, Thoraxcenter, Erasmus University Medical Center, 3000 CB Rotterdam, The Netherlands; yzsener@yahoo.com.tr; 4 Department of Pathophysiology, Faculty of Medicine, University of Sarajevo, 71000 Sarajevo, Bosnia and Herzegovina; almir.fajkic@mf.unsa.ba; 5 Department of Internal Medicine, Division of Nephrology and Dialysis, University Hospital of Split, 21000 Split, Croatia; mavuckovic@kbsplit.hr (M.V.); josiparadic1973@gmail.com (J.R.); 6 Department of Physiology, School of Medicine, University of Mostar, 88000 Mostar, Bosnia and Herzegovina; antonio.markotic@mef.sum.ba; 7 Department of Internal Medicine, General Hospital “Dr. Josip Benčević” Slavonski Brod, Andrije Štampara 42, 35000 Slavonski Brod, Croatia; mirji.stanic@gmail.com; 8 Institute for Clinical Medical Research and Education, Sestre Milosrdnice University Hospital Center, School of Medicine Catholic University of Croatia, 10000 Zagreb, Croatia; potocnjak.ines@yahoo.com; 9 Hospital for Medical Rehabilitation of Heart and Lung Diseases and Rheumatism ‘Thalassotherapia-Opatija’, 51410 Opatija, Croatia; marija.rogoznica@gmail.com; 10 Internal Medicine Department, School of Medicine, University of Split, 21000 Split, Croatia; 11 Department of Internal Medicine, Division of Rheumatology, Allergology and Clinical Immunology, Center of Excellence for Systemic Sclerosis in Croatia, University Hospital of Split, 21000 Split, Croatia

**Keywords:** disease-modifying antirheumatic drugs, rheumatoid arthritis, JAK inhibitors, JAK-STAT pathway, pain modulation

## Abstract

Pain remains one of the most burdensome symptoms in rheumatoid arthritis (RA), often persisting despite inflammatory remission and profoundly impairing quality of life. This review aimed to evaluate the clinical efficacy and mechanistic pathways by which Janus kinase (JAK) inhibitors alleviate RA-related pain. Evidence from randomized clinical trials demonstrates that JAK inhibitors have demonstrated rapid and significant pain relief, often exceeding that of methotrexate or biologic DMARDs. Improvements in patient-reported pain scores seem to typically emerge within 1–2 weeks and are sustained over time. Beyond anti-inflammatory effects, JAK inhibitors modulate central sensitization and nociceptive signaling by attenuating IL-6 and GM-CSF activity, reducing astrocyte and microglial activation, and downregulating nociceptor excitability in dorsal root ganglia and spinal pathways. Preclinical models further suggest that JAK inhibition interrupts neuroimmune feedback loops critical to chronic pain maintenance. Comparative and network meta-analyses consistently position JAK inhibitors among the most effective agents for pain control in RA. However, individual variability in response, partly due to differential JAK-STAT activation and cytokine receptor uncoupling, underscores the need for biomarker-guided treatment approaches. JAK inhibitors represent a mechanistically distinct and clinically impactful class of therapies that target both inflammatory and non-inflammatory pain in RA. Their integration into personalized pain management strategies offers a promising path to address one of RA’s most persistent unmet needs.

## 1. Introduction

Rheumatoid arthritis (RA) is a persistent, systemic autoimmune disease characterized by inflammation of the synovium, loss of joint function, and chronic pain [1,2]. With the introduction of disease-modifying antirheumatic drugs (DMARDs) and biologic therapy, certain methods of treatment have improved [3]. However, pain is still one of the most common and distressing symptoms for most patients, even in the absence of inflammation [4]. A common practice in the treatment of RA is based on controlling inflammation and damage to tissue structures, resulting in a surprisingly high number of patients experiencing pain despite achieving remission [5].

RA causes pain through both inflammatory and non-inflammatory mechanisms [6,7,8]. Inflammatory pain is caused by inflammation of the synovium, which activates nociceptors and leads to peripheral sensitization and pain. Chronic inflammation can also alter peripheral nerve function, which exacerbates nociceptive signals [6]. Non-inflammatory pain involves central sensitization and increased central nervous system (CNS) responsiveness due to neuroinflammation and synaptic plasticity. Pro-inflammatory cytokines and autoantibodies interfere with pain processing in the CNS and cause widespread hypersensitivity [9,10]. Pain may persist despite control of inflammation, reflecting the complex interplay of central sensitization and neuro-immune interactions. This duality necessitates multimodal therapies targeting both peripheral inflammation and central mechanisms [6,11].

In RA, the Janus kinase-signal transducer and activator of transcription (JAK-STAT) pathway is an essential intracellular signaling cascade. Cytokines attach to cell surface receptors, triggering Janus kinases (JAKs)—specifically JAK1, JAK2, JAK3, and tyrosine kinase 2 (TYK2). These kinases phosphorylate and recruit signal transducers and activators of transcription (STATs), which move to the nucleus to control gene expression. This process ultimately drives the generation of pro-inflammatory cytokines and prolongs chronic inflammation in RA [12,13].

Currently available JAK inhibitors (JAKi) are tofacitinib, baricitinib, upadacitinib, and filgotinib (Table 1). They have been shown to be effective in reducing disease activity and halting radiographic progression. Notably, it also appears that early pain relief occurs before inflammatory indicators return to baseline—even in the first 24 h [14,15], which may indicate a function in attenuating central pain processing. Clinically significant efficacy in this regard is seen after only two weeks, with the greatest effect occurring after three months [14]. Patients treated with baricitinib experienced greater and more consistent pain relief at one to four weeks of therapy compared to those treated with placebo or adalimumab [16]. Current evidence suggests that JAKi modulate central pain processing pathways by interfering with cytokines such as interleukin-6 (IL-6) and granulocyte-macrophage colony-stimulating factor (GM-CSF), which activate JAK/STAT signaling associated with central sensitization [14].

By integrating clinical data and molecular perspectives, this paper will explore the growing importance of JAKi in the treatment of RA-related pain.

## 2. Pathophysiology of Pain in RA and the JAK-STAT Pathway

Pain in RA involves both inflammatory and non-inflammatory mechanisms. While traditionally linked to synovial inflammation, increasing evidence highlights a multifactorial pathophysiology. The pathophysiology of pain in RA involves several interrelated mechanisms. Key mechanisms include synovial fibroblast activity, dorsal root ganglia (DRG) alterations, central sensitization, neuro-immune interactions, and JAK-STAT signaling.

Synovial fibroblasts play a key role by promoting the growth of pain-sensitive neurons, such as those expressing calcitonin gene-related peptide (CGRP), into areas of synovial overgrowth. These cells, for instance, have been shown to contribute to pain even without active inflammation. Studies have shown that supernatants from cultured fibroblasts can stimulate branching in DRG neurons, suggesting a direct contribution to sensory nerve expansion [17]. Moreover, when activated by tumor necrosis factor α (TNF-α), these fibroblasts can further enhance peripheral sensitization and pain [17,18].

Changes within the DRG are significant, as these neurons are central to pain transmission. Transcriptomic studies of DRG in RA patients reveal gene expression changes related to inflammation and nerve growth, which may enhance pain sensitivity [19]. Nociplastic pain—driven by altered pain processing within the CNS—is another key concept. This type of pain can persist even after inflammation resolves, pointing to mechanisms of central sensitization [20].

Peripheral factors, including interactions between immune cells, autoantibodies, and peripheral nerves, also sustain chronic pain in RA through neuro-immune pathways. Additionally, the high mobility group box-1 (HMGB1) protein contributes to pain by activating both inflammatory and nociceptive pathways [21,22].

Central sensitization—a heightened sensitivity of the CNS to pain signals—also contributes to RA-related pain, often persisting after inflammation has subsided. Neuro-immune interactions, particularly those involving crosstalk between immune and nervous system components, are further modulated by the JAK-STAT pathway and play a role in pain persistence [23,24].

Taken together, the JAK-STAT signaling pathway emerges as a central player in RA pain, influencing peripheral nerve activity, central pain processing, and immune-nervous system communication.

Building on this, significant effort has been made to elucidate how these processes converge in rheumatic diseases, with the JAK–STAT pathway emerging as a pivotal component [24]. Beyond its role in inflammation, inhibition of this pathway may provide pain relief by modulating central pain processing and sensory neuron excitability. Central sensitization, present in almost 40% of RA patients [25,26], reflects abnormal nociceptive input processing largely driven by neuro-immune interactions and inflammatory signaling within the CNS [27]. The neuroinflammatory environment is accompanied by activation of microglial cells and astrocytes, with further production of cytokines and neurotrophic factors contributing to increased excitability and chronic pain [24].

The JAK-STAT signaling pathway is a well-conserved mechanism that plays a central role in regulating immune function, blood cell formation, and cellular development. It is activated when cytokines or growth factors bind to specific receptors on the cell surface, triggering the associated Janus kinases (JAK1, JAK2, JAK3, and TYK2) [28,29].

Once activated, TYK phosphorylates tyrosine residues on the receptor, creating docking sites for STAT proteins. These STATs are then phosphorylated, allowing them to form dimers and move into the nucleus, where they act as transcription factors to regulate genes involved in immune responses, cell proliferation, and differentiation [30].

Under normal physiological conditions, this pathway is tightly controlled to ensure appropriate responses to external stimuli. However, when dysregulated—often due to mutations—it can contribute to the development of various diseases. For instance, gain-of-function mutations in JAK2 are frequently found in myeloproliferative disorders, while alterations in other JAK family members have been linked to immune imbalance and hematologic malignancies [31].

Disruptions in the JAK-STAT signaling pathway are involved in a wide range of conditions, particularly hematologic cancers, autoimmune diseases, and immunodeficiencies. In hematologic malignancies, somatic gain-of-function mutations in JAKs—especially JAK2—are common. The JAK2 V617F mutation is a defining feature of disorders such as polycythemia vera, essential thrombocythemia, and myelofibrosis, and it has also been reported in acute leukemias. Moreover, aberrant JAK1/2 signaling contributes to the development of leukemias and lymphomas [32,33].

Autoimmune diseases such as RA, inflammatory bowel disease, and systemic lupus erythematosus are also linked to JAK-STAT dysregulation. The therapeutic success of JAKi in these conditions further highlights the pathway’s clinical relevance [34].

In the context of immunodeficiencies, inherited mutations in JAK3 are associated with severe combined immunodeficiency (SCID), while PBMC mutations are linked to autosomal recessive hyperimmunoglobulin E syndrome. These genetic defects impair immune cell development and function, emphasizing the critical role of JAK-STAT signaling in maintaining immune system integrity [35,36].

The JAK-STAT signaling pathway plays an essential role in developing RA by transmitting signals from cytokines involved in inflammation and immune regulation. When cytokines bind to their receptors, they activate JAKs, which phosphorylate STAT proteins. These STATs then dimerize and move into the nucleus, influencing gene expression [37,38].

Within this system, several specific pathways have been identified as particularly relevant to disease development and progression (Table 2):

1. IL-6/JAK/STAT3 Axis: IL-6 is a key pro-inflammatory cytokine in RA. It activates JAK1, JAK2, and TYK2 kinases, which in turn phosphorylate the STAT3 protein. Elevated levels of phosphorylated STAT3 (pSTAT3) have been found in RA patients’ peripheral blood mononuclear cells (AP2) and are correlated with markers of inflammation such as erythrocyte sedimentation rate (ESR), C-reactive protein (CRP), and Disease Activity Score (DAS28). This axis is not only a marker of disease activity but also a therapeutic target, with JAKi demonstrating the ability to modulate its effects [39].
2. Predominant Role of JAK1: JAK1 is critically involved in signaling for several pro-inflammatory cytokines, including IL-6 and interferons. It contributes to synovial inflammation and bone remodeling, making JAK1-selective inhibitors an attractive treatment strategy in RA. Targeting JAK1 specifically may help reduce inflammation while minimizing side effects associated with broader JAK inhibition [40].
3. Impaired JAK/STAT Activity in Some Patients: Despite elevated serum cytokine levels, certain RA patients exhibit reduced JAK/STAT pathway activation. This paradox may explain why some patients respond poorly to cytokine-targeted therapies. Evaluating STAT protein phosphorylation could help identify individuals at higher risk of treatment resistance [41].
4. Role of SOCS Proteins: Suppressors of cytokine signaling (SOCS) proteins, such as SOCS2, function as natural inhibitors of the JAK-STAT pathway. In RA, reduced expression of SOCS2 may allow unchecked pathway activation, contributing to chronic inflammation and ongoing joint damage [42].

Patients with active RA, particularly those with elevated levels of pro-inflammatory cytokines like IL-6, are commonly affected by disruptions in the JAK-STAT signaling pathway. Interestingly, not all patients follow the expected pattern—some show reduced activation of the JAK/STAT pathway despite having high circulating cytokine levels, which may help explain why certain individuals do not respond well to cytokine-targeted therapies [43].

Recent findings highlight that IL-6-driven STAT3 activation is especially pronounced in CD4+ T cells during the early phases of RA, and this is particularly evident in patients who test negative for anticitrullinated peptide antibodies (ACPA)-negative [44]. This suggests that heightened IL-6/STAT3 signaling could be a distinguishing feature of early or seronegative RA subtypes. Moreover, how this pathway behaves can vary across different immune cell populations, some of which show ongoing STAT3 activation that appears to mirror IL-6 levels in the blood [39].

The observation that some patients have abundant cytokines but muted downstream signaling raises the possibility of a disconnect, or “uncoupling,” between cytokine availability and cellular response. Several factors might contribute to this, including genetic predispositions, receptor fatigue or desensitization, and alterations in the molecules that transmit signals inside cells [41].

Recognizing these subtle but important differences in pathway activity is key to understanding why certain patients do not benefit from standard treatments. It also underscores the importance of moving toward more personalized approaches in managing RA. Together, these pathways illustrate the complexity of JAK-STAT signaling in RA and highlight its potential as a therapeutic target. Modulating these mechanisms, particularly through selective JAK inhibitors, offers a promising approach to managing inflammation and altering the course of the disease.

## 3. JAK Inhibitors and Pain: Mechanisms Beyond Inflammation

As outlined in Section 2, the pathophysiology of RA-related pain involves both peripheral inflammatory and central sensitization mechanisms, with the JAK–STAT pathway serving as a critical signaling hub. Building on this mechanistic framework, the following section reviews experimental and clinical evidence demonstrating how JAK inhibition modulates these pain pathways. Particular attention is given to its effects on central sensitization, neuroinflammation, sensory neuron excitability, and clinically relevant pain outcomes in RA patients. Choy and Calabrese summarized preclinical findings that support the dominant role of IL-6 in the pain mechanism in RA [45]. Cells in the spinal cord, e.g., neurons and glial cells, and DRG express gp130, which makes them susceptible to IL-6 signaling [46]. Vazquez et al. showed that spinal IL-6 amplifies the arthritic pain in a murine model as joint inflammation significantly increases spinal release of IL-6, contributing to central sensitization and hyperalgesia [47]. Soluble gp130, which abolishes trans-signaling, hampered the development of spinal hyperexcitability, suggesting that IL-6 is pivotal for its generation [47]. Furthermore, it is suggested that IL-6 is capable of inducing peripheral sensitization. Brenn et al. analyzed the electrophysiological records from afferent fibers after applying IL-6 alone or with soluble IL-6 receptor (sIL-6R) by intraarticular injections in rats [48]. This caused the sensitization of the nociceptive C-fibers to mechanical stimuli. Again, concomitant administration of soluble gp130 prevented the development of sensitization [48].

Both IL-6 signaling pathways, classic and trans-signaling, use gp130 and JAK to transduce signals in the cells, and JAKi might alleviate pain by affecting peripheral and central nociception [49]. Makabe et al. showed that baricitinib, a JAK1/2 inhibitor, alleviates inflammatory and neuropathic pain in a mouse arthritis model through modulation of IL-6/JAK/STAT3 and expression of colony-stimulating factor 1 (CSF-1) in neurons of DRG [50]. Baricitinib also reduced the proliferation of microglia and astrocytes in the spinal dorsal horn, which are related to the pathophysiology of neuropathic pain [50,51]. Additionally, baricitinib can diminish the direct sensitizing effect of IL-6 + sIL-6R on nociceptive C-fibers and prevent neuronal hyperexcitability [52]. A novel study by Simon et al. shed new light on the antinociceptive effect of baricitinib [53]. The authors found that not only is the JAK/STAT pathway affected by baricitinib, but baricitinib also inhibits the adaptor protein-2 (AP2) associated kinase 1 (AAK1) signaling. AAK1 inhibition effectively reversed the mechanical hypersensitivity. Baricitinib decreased STAT3 phosphorylation in joints and DRG, reversed mechanical hypersensitivity and hyperinnervation of synovia in the late phase, and dose-dependently suppressed neuronal excitability in vitro [53]. Collectively, these findings indicate that pain reduction by JAKi may be, at least in part, exerted through direct modulation of neuronal activity.

The function of microglia and astrocytes, which are essential factors in neuroinflammatory processes, is also affected by JAKi [44]. Ruxolitinib significantly reduced the lipopolysaccharide-stimulated activation of microglia and astrocytes and the expression of major pro-inflammatory cytokines [54]. In a mouse model of RA, baricitinib decreased the activity of microglia in the area postrema brain region with inhibition of STAT3 phosphorylation and improvement in behavioral outcomes such as food intake [55]. Furthermore, the gp130/JAK/STAT3 signaling pathway mediates the proliferation of astrocytes, which, besides microglia, are found to be a major component in the maintenance of neuropathic pain [56]. Disruption of this pathway by JAK inhibitor AG490 reduced the proliferation of dorsal horn astrocytes and relieved tactile allodynia induced by nerve injury in rats [57]. There is also evidence that JAK/STAT signaling modulates chronic pain through interaction with N-methyl-D-aspartate (NMDA) receptors in the spinal dorsal horn. These receptors contribute to pain transmission and peripheral and central sensitization [58]. AG490 significantly attenuates leptin-induced NMDA currents that play a role in thermal hyperalgesia and mechanical allodynia in dorsal horn neurons [59].

However, JAK and JAK/STAT pathway activation is not exclusively related to IL-6. Several other cytokines and factors, either pro- or anti-nociceptive, stimulate this pathway [60]. GM-CSF also signals through the JAK2 molecule and contributes to arthritic pain, mechanical hyperalgesia, and nociceptive signaling in RA [61,62]. The downregulation of the JAK2/STAT3 pathway inhibits the GM-CSF-induced upregulation of sodium voltage-gated channels and alleviates hyperalgesia in vivo [63].

A summary of JAK inhibitors’ mechanism affecting pain pathways and sensitization is given in Table 3.

### Comparison with TNF-α Inhibitors: Differences in Central vs. Peripheral Pain Modulation

**Figure 1:**
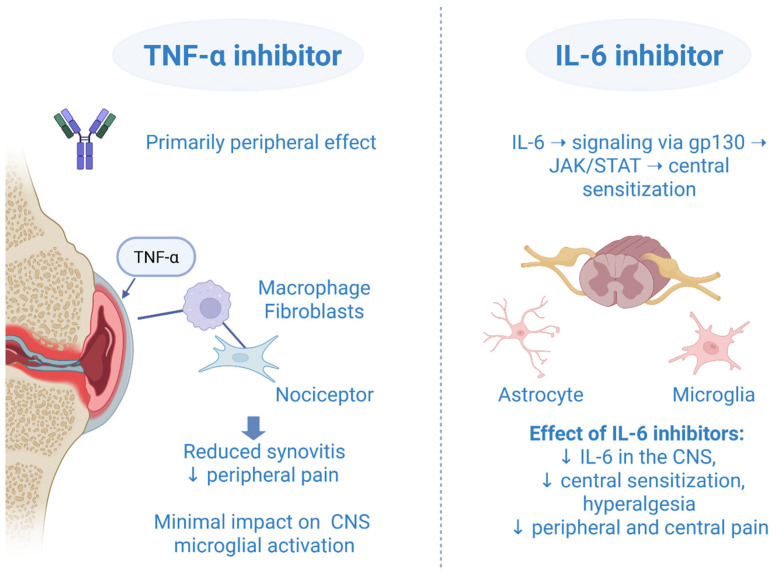
Comparative mechanisms of TNF-α and IL-6 inhibitors in central and peripheral pain modulation. Created in BioRender. Vučković, M. (2025) https://biorender.com/dxuprpb (accessed on 1 October 2025).

Crosstalk, overlapping, and the interplay effect of IL-6 inhibitors and TNF-α inhibitors are all well-used terms that describe their relationship in a complex neuroinflammatory mechanism of action. It is not possible to fully and accurately describe the role of an IL-6 inhibitor or TNF-α inhibitor without involving the other one, as each has the potential to impact the other through more than one signal pathway. At the molecular level, it is found that almost 55% (287 genes) of total IL-6/sIL-6R-regulated genes are common to those of TNF-α [66]. The inhibition of IL-6 suppressed the elevation of TNF-α, IL-1, and IL-6 messenger RNA in the spinal cord and inhibited the release of TNF-α and IL-1 protein into cerebrospinal fluid [67]. Given that the TNF-α inhibitors were available earlier on the market, the clinical effect of IL-6 inhibitors has been estimated after TNF-α inhibitor failure. In a RADIATE randomized controlled study, tocilizumab compared to placebo showed a significant improvement in pain, disability, and fatigue in patients with RA refractory to TNF-α inhibitors [68]. Although a sarilumab monotherapy was associated with a better improvement of patient-reported outcomes (including pain) compared to adalimumab monotherapy in a head-to-head MONARCH trial, we should not make the pitfall of noting that IL-6 inhibitors are more important or even better than TNF-α inhibitors [69]. Both drugs are key players in peripheral and central pain modulation, and they cannot be evaluated separately without mentioning the other one. They showed synergistic effects in pain processing. TNF-α primarily contributes to peripheral inflammation and nociceptive signaling, while IL-6, through the gp130/JAK/STAT pathway, plays a major role in central sensitization. The convergence and synergistic actions of these cytokine pathways form the conceptual basis for the use of JAKi in modulating pain responses in RA (Figure 1). Moreover, in 2023 it was announced that a bispecific nanobody compound, which blocked human TNF-α and IL-6, effectively inhibited disease-relevant pathways in vitro, supporting further development of this TNF/IL-6 nanobody as a therapy for RA [70].

## 4. Clinical Evidence: JAK Inhibitors for Pain Relief

RA is an autoimmune disease characterized by chronic inflammation, pain, joint deformity, disability [71], and lowered quality of life (QOL), often requiring pharmacotherapy. Pain is one of the most prevalent complaints in patients with RA. In clinical practice, it is essential to select the correct pharmacotherapy [72].

Over the past decades, several conventional synthetic disease-modifying antirheumatic drugs (csDMARDs) have been introduced into clinical practice for the management of RA: injectable gold in the 1930s, hydroxychloroquine in the 1950s, azathioprine in the 1960s, sulfasalazine and methotrexate (MTX) in the mid-1980s, and leflunomide in the late 1990s [73,74]. Among these, only gold salts are no longer in clinical use. Although MTX represented a major therapeutic breakthrough, its efficacy is not universal; more than 30% of patients discontinue treatment within the first year due to adverse events or insufficient clinical response [75].

The advent of biologic DMARDs (bDMARDs) in the past 25 years has been considered a milestone, offering more targeted and potentially less toxic immunomodulation [14]. TNF-α inhibitors were the first bDMARDs approved by the US Food and Drug Administration for RA, introduced sequentially as follows: etanercept (1998), infliximab (1999), adalimumab (ADA) (2002), certolizumab pegol (2009), and golimumab (2009) [76]. Subsequently, agents targeting T-cell co-stimulation, as well as B-cell depletion via CD20-directed monoclonal antibodies, became available in the mid-2000s. In addition, IL-6R inhibitors tocilizumab and sarilumab, both humanized monoclonal antibodies, were approved in 2010 and 2017; these agents exert their effects by inhibiting IL-6-mediated signaling [77]. Despite these therapeutic advances, a considerable proportion of patients with RA continue to experience suboptimal disease control, secondary loss of efficacy, or intolerance to currently available bDMARDs.

JAKi are classified within the group of targeted synthetic disease-modifying antirheumatic drugs (tsDMARDs). The first JAKi created and introduced to the market for the treatment of RA was tofacitinib. It was developed in the middle of the 1990s, approved by the Food and Drug Administration (FDA), and introduced on the market in 2012 [78]. After more than four years of post-marketing safety monitoring in North America, tofacitinib was ultimately authorized by the European Medicines Agency (EMA) in March 2017. Baricitinib was approved by the FDA in May 2018 and by the EMA in December 2016. Upadacitinib received approval from the FDA in August 2019 and from the EMA in December 2019 [14]. Filgotinib was approved in Europe and Japan in September 2020 [79].

JAKi are targeted oral agents with a mechanism of action broadly comparable to TNF-α inhibitors and a similar safety profile [14]. Their oral administration renders them particularly attractive from the patient perspective. Notably, JAKi have been associated with rapid pain relief, often preceding normalization of inflammatory markers, which constitutes an additional therapeutic advantage of this drug class [14]. Another important feature of JAKi is their relatively short half-life. This pharmacokinetic property facilitates more rapid drug clearance, which may be of particular relevance in patients at increased risk of infectious complications. Such considerations are especially pertinent in the perioperative setting, where timely withdrawal of immunosuppressive therapy may reduce postoperative infection risk. Moreover, a shorter half-life provides clinicians with greater flexibility in therapeutic management, allowing for more rapid adjustments in the event of adverse reactions or changes in disease activity [80].

In large international randomized clinical trials (RTCs), pain relief is usually one of the primary or secondary endpoints or components of scales used, such as the American College of Rheumatology (ACR), DAS28-CRP, Health Assessment Questionnaire-Disability Index (HAQ-DI), physician’s global assessment of disease activity, patient’s global assessment, and pain [81].

Identified tools and scales used in RTCs and observational studies for assessment of RA patients are:

The ACR criteria is a tool used to evaluate the efficacy of treatments for RA and is often the primary endpoint (indicated as ACR 20, 50, or 70). The ACR measures improvement in the count of tender or swollen joints and improvement in at least three of the following: patient pain scale, patient and physician global assessment of disease activity, disability/functional questionnaire, and acute phase reactant (ESR or CRP); HAQ-DI score; DAS28-CRP (describes severity of RA using clinical [tender joint count, swollen joint count], global health [as reported on visual analog scale (VAS)], and CRP); DAS28-4/ESR; Simplified Disease Activity Index (SDAI) score; Patient Assessment on Pain VAS; Pain DETECT questionnaire; Short-form McGill Pain questionnaire; Chronic pain assessment questionnaire; Physician’s global assessment of disease activity; Patient Global Assessment (PtGA); 36-Item Short Form Health Survey Physical Component Score (SF-36 PCS); Fatigue (functional assessment of chronic illness therapy-fatigue (FACIT-F)); Clinical Disease Activity Index (CDAI); Individual ACR core set parameters; Tender Joint Count 68; Swollen Joint Count 66; Acute phase reactants (ESR, CRP concentration); The Hospital Anxiety and Depression Scale (HADS); Beck Depression Inventory (BDI); The State-Trait Anxiety Inventory (STAI).

Vergne-Salle et al. [82], in a cross-sectional study, concluded that 38.4% of patients continue to experience moderate to severe pain despite a high proportion (more than 80%) being on biological therapy. In this study, authors conducted an assessment combining quantitative and qualitative aspects; patients completed a chronic pain assessment questionnaire, the HAQ, depression and anxiety scales, and DAS28 and ESR. The authors identified a positive correlation between pain and anxiety/depression and also between pain and disease activity. The impact of pain on daily life was the most significant on working life, followed by the impact of pain on mood and sleep. The limitation of such cross-sectional studies is that they do not assess fluctuations in pain over time [82]. Sung et al. [83] published a network meta-analysis since several clinical trials compared the efficacy and safety of tofacitinib, baricitinib, upadacitinib, and filgotinib against MTX in DMARDs-naive RA patients. However, effectiveness and safety are uncertain, as there were no clinical trials directly comparing JAKi [84]. They concluded that tofacitinib, baricitinib, upadacitinib, and filgotinib were effective pharmacotherapy options for DMARD-naive RA patients [83]. However, they suggest a difference in efficacy and safety among JAKi [83]. Cai et al. [72] published a systematic review and network meta-analysis of RTCs on the comparative efficacy of five approved JAKi as monotherapy and combination therapy in patients with moderate-to-severe active RA. Their comprehensive research showed that all JAKi were more effective than placebo in ACR20, ACR50, ACR70, and DAS28-CRP < 2.6 at 12 weeks [72]. Furthermore, they were also more effective than csDMARDs or placebo in ACR20, ACR50, ACR70, and DAS28-CRP < 2.6 at 24 weeks [72]. Lee YH et al. [85] conducted a network meta-analysis and included information from RTC that examined remission DAS28-CRP by tofacitinib, baricitinib, upadacitinib, filgotinib monotherapy, and MTX in DMARDs-naive RA patients. Their research suggests that upadacitinib seems to be one of the most effective interventions for achieving remission in DMARDs-naive patients with RA based on the comparative analysis, followed by tofacitinib, baricitinib, filgotinib, and MTX. A summary of clinical evidence from trials is given in Table 4.

### 4.1. Baricitinib

As published by Fleischmann et al., the RA-BEGIN study was a randomized, double-blind, phase 3 study that compared baricitinib, baricitinib + MTX, and MTX in patients with active RA [86]. RA-BEGIN concluded that baricitinib alone or in combination with MTX demonstrated superior efficacy (superior to MTX at week 24, with a higher ACR20 response rate: 77% vs. 62%) [86]. Significant improvement was observed in tender and swollen joints, hsCRP level, pain, and physician’s and PtGA [86]. Taylor et al. [87] published a post hoc analysis of the RA-BEGIN study in which they assessed the magnitude, speed, and maintenance of pain improvement in early RA receiving baricitinib, baricitinib and MTX, or MTX over 1 year, as well as cumulative pain and QOL. The authors concluded that patients treated with baricitinib had significantly greater and more rapid pain relief, 9–10 additional weeks of limited to no pain, and clinically meaningful improvements in physical health [87]. Furthermore, in this study, the authors analyzed PtGA and SF-36 PCS outcomes; baricitinib, alone or in combination gained wellness measured by PtGA and 5–7 additional weeks with change in SF-36 PCS ≥ 5 vs. MTX over 1 year [87]. They explain in their paper that physicians focus on treating the inflammation in RA; however, pain is often the primary concern for patients [87]. Another important patient-reported outcome in RA is PtGA, the patient’s perception of disease activity, for which pain is one of the main components. The SF-36 scale is a validated patient-reported outcome (PRO) to evaluate HRQoL [87]. In the phase 3 study RA-BEACON of baricitinib (2 or 4 mg daily) in patients with refractory RA, Genovese et al. [88] concluded that baricitinib at 4 mg was associated with clinical improvement at 12 weeks. Significantly more patients receiving baricitinib 4 mg than those on placebo had an ACR20 response at week 12 (55% vs. 27%) [88]. Differences between the baricitinib 4 mg group and the placebo were also significant for the HAQ-DI score and the DAS28-CRP but not for an SDAI score of 3.3 or less [88]. Schiff et al. [89] published a study evaluating patient-reported outcomes in a phase 3 study of baricitinib as monotherapy or combined with MTX in patients with active RA. The authors conclude that baricitinib (alone or in combination), when used as initial therapy, showed improvement compared to MTX in most PRO measures [89]. Fautrel et al. [90] published exploratory analyses from RA-BEAM, a phase 3 study, of the effect of baricitinib and adalimumab on reducing pain and improving function. They found that baricitinib 4 mg improved pain and physical function in well-controlled RA patients, explaining it could have effects beyond immunomodulation [88].

### 4.2. Tofacitinib

The phase 3 study ORAL Start by Lee EB et al. [91] on monotherapy tofacitinib versus MTX in RA reported findings of a randomized, double-blind study. Among the patients receiving tofacitinib, 25.5% in the 5 mg group and 37.7% in the 10 mg group had an ACR 70 response at month 6, as compared with 12% of patients in the MTX group (*p* < 0.001) [85]. Patients in both tofacitinib groups reported greater pain and disease activity reductions at month 6 [85]. In a randomized phase 3 trial, ORAL STEP by Burmester et al. investigated tofacitinib (tofacitinib at 5 mg or 10 mg twice per day—BID) in combination with MTX in patients with active RA with an inadequate response to TNF inhibitors [92]. The study concluded that tofacitinib with MTX had rapid and clinically meaningful improvements in RA symptoms, signs, and physical function over 6 months [92]. At month 3, ACR20 response rates were 41.7% and 48.1% for tofacitinib 5 mg and 10 mg BID versus 24.4% for placebo; improvements from baseline in HAQ-DI were −0.43 and −0.46–0.18, respectively; and DAS28 < 2.6 rates were 6.7%, 8.8%, and 1.7%, respectively [92]. Fleischmann et al. [93], in the ORAL Solo study of tofacitinib in RA, concluded that tofacitinib monotherapy was associated with reductions in RA symptoms and signs and improvement in physical function; patients also had significant decreases in pain and fatigue levels.

### 4.3. Upadacitinib

Van Vollenhoven et al. [94], in the SELECT-EARLY trial, evaluated the effect of upadacitinib as monotherapy in patients with predominantly early RA (naive to or had limited exposure to MTX). The authors concluded that patients receiving upadacitinib experienced significant clinical improvements and patient-reported outcomes compared to patients receiving MTX. Significant and clinically meaningful improvements in multiple patient-reported outcomes were recorded for upadacitinib versus MTX [94]. A double-blind, phase 3, RCT, SELECT-BEYOND by Genovese et al. [81], evaluated the efficacy and safety of upadacitinib in patients with active RA refractory to bDMARDs. The study concluded that doses of 30 and 15 mg of upadacitinib led to rapid and significant improvements compared with a placebo over 12 weeks [81]. At week 12, ACR20 was achieved by 65% and 56% of patients receiving upadacitinib 15 mg and 30 mg, respectively, compared with 28% receiving placebo [81]. For each component of the ACR (including the patient’s GA and pain), patients receiving upadacitinib had significantly better scores. Furthermore, improvements continued through week 24. Patients treated with upadacitinib improved physical function and the severity and duration of morning stiffness. The authors showed that upadacitinib could achieve significant, rapid improvement in clinical, functional, and patient-reported outcomes [81].

### 4.4. Filgotinib

In the study FINCH 2 by Genovese et al. [84], on the effect of filgotinib compared to placebo on clinical response in patients with moderate to severe RA refractory to DMARDs. In this phase 3 RCT, authors conclude that among patients with active RA who had an inadequate response or intolerance to one or more bDMARDs, filgotinib achieved a greater clinical response at week 12 [84]. The patient’s pain, ptGA, and physician’s GA were measured on VAS [84]. In the study FINCH 3, a phase 3 RCT by Aletaha et al. [95] evaluating the efficacy and safety of filgotinib in MTX-naive patients with RA, the authors showed greater pain and physical function improvement. Treatment with filgotinib versus MTX monotherapy was associated with similar or greater improvement in physical function and patient-assessed pain. Patient-assessed pain was further decreased from baseline at weeks 24 and 52 in patients treated with filgotinib plus MTX or filgotinib monotherapy at week 24 [95]. Atsumi et al. [71], in the FINCH 3 study of long-term safety and efficacy, concluded that efficacy was maintained through week 52 and longer.

**Table 4:** Summary of clinical evidence from trials.

JAK Inhibitors	Authors:	Drugs Compared	Study	Concluded
Baricitinib	Genovese MC et al. (2016) [88]	baricitinib (2 or 4 mg daily) and placebo	RA-BEACON	baricitinib at 4 mg was associated with clinical improvement at 12 weeks
	Fleischmann R et al. (2017) [86]	baricitinib, baricitinib + MTX, and MTX	RA-BEGIN	baricitinib alone or in combination with MTX demonstrated superior efficacy
	Schiff M et al. (2017) [89]	baricitinib as monotherapy or combined with MTX		baricitinib (alone or in combination), when used as initial therapy, showed improvement compared to MTX in most PRO measures
	Fautrel B et al. (2019) [90]	baricitinib and adalimumab, placebo	RA-BEAM (exploratory analyses)	Baricitinib 4 mg provided enhanced improvement in pain and physical function
	Taylor PC et al. (2022) [87]	baricitinib, baricitinib + MTX, and MTX over 1 year	RA-BEGIN (post hoc analysis)	patients treated with baricitinib had significantly greater and more rapid pain relief, 9–10 additional weeks of limited to no pain, and clinically meaningful improvements in physical health
Tofacitinib	Fleischmann R et al. (2012) [93]	tofacitinib monotherapy, Placebo	ORAL Solo	tofacitinib monotherapy was associated with reductions in RA symptoms and signs and improvement in physical function
	Burmester GR et al. (2013) [92]	tofacitinib (5 mg or 10 mg BID) combined with MTX	ORAL STEP	tofacitinib with MTX had rapid and clinically meaningful improvements in RA symptoms, signs, and physical function over 6 months
	Lee EB et al. (2014) [91]	tofacitinib, baricitinib, upadacitinib, filgotinib monotherapy, and MTX	ORAL Start	Treatment with tofacitinib, baricitinib, upadacitinib, filgotinib achieved a significantly higher remission rate than MTX
Upadacitinib	Genovese MC et al. (2018) [81]	upadacitinib compared with a placebo	SELECT-BEYOND	upadacitinib led to rapid and significant improvements compared with a placebo over 12 weeks
	Van Vollenhoven R et al. (2020) [94]	upadacitinib as monotherapy, methotrexat	SELECT-EARLY	patients receiving upadacitinib experienced significant clinical improvements and PROs compared to patients receiving MTX
Filgotinib	Genovese MC et al. (2019) [84]	filgotinib compared to placebo	FINCH 2	patients with active RA who had an inadequate response or intolerance to one or more bDMARDs, filgotinib achieved greater clinical response at week 12
	Aletaha D et al. (2021) [95]	filgotinib versus MTX, filgotinib plus MTX	FINCH 3 (post hoc analysis)	showed greater pain and physical function improvement
	Atsumi T et al. (2023) [71]	filgotinib, filgotinib plus MTX or MTX	FINCH 3 (Long-term safety)	efficacy was maintained through week 52 and longer
Network meta-analysis	Sung YK et al. (2021) [83]	tofacitinib, baricitinib, upadacitinib, and filgotinib versus methotrexate	network meta-analysis	tofacitinib, baricitinib, upadacitinib, and filgotinib were effective pharmacotherapy options for DMARDs-naive RA patients
	Lee YH et al. (2023) [85]	tofacitinib, baricitinib, upadacitinib, filgotinib monotherapy, and MTX	network meta-analysis	upadacitinib seems to be one of the most effective interventions for achieving remission
	Cai W et al. (2024) [72]	five approved JAK inhibitors as monotherapy and combination therapy	network meta-analysis	all JAK inhibitors were more effective than placebo

MTX—methotrexate. PRO—patient-reported outcomes.

## 5. Head-to-Head Comparisons and Indirect Evidence

As summarized in Section 2, RA-related pain arises from both peripheral inflammatory drivers and central sensitization mechanisms. With this pathophysiological background in mind, the limited impact of traditional RA therapies on these pain pathways has raised interest in whether JAKi, by targeting a broader range of cytokines and signaling cascades, might offer superior pain control in patients with long-standing disease [96,97]. While JAKi’s effectiveness in reducing disease activity in RA is well-documented, their role in managing RA-related pain has also garnered significant attention. This is particularly relevant, as pain remains a persistent concern for RA patients, often continuing even when clinical signs of inflammation are under control. There are several studies comparing different JAKi (tofacitinib, upadacitinib, baricitinib, peficitinib, filgotinib, and decernotinib) with placebo, csDMARDs, or biologic agents in terms of different clinical endpoints in patients with RA. However, there are no studies comparing different JAKi with each other in RA cases. Recently published network meta-analyses provide data about comparing JAKi in RA-related outcomes [72,98].

Surface under the cumulative ranking curve (SUCRA) values of JAKi at 24 weeks of the treatment were compared to each other in terms of clinical efficacy scores used in RA, including ACR20, ACR50, and ACR70. The SUCRA value of upadacitinib 15 mg + csDMARD (81.1%) was the largest for the ACR20 score, while the SUCRA values of baricitinib 4 mg + csDMARD (85.4%) and upadacitinib 30 mg (92.4%) were the highest for the ACR50 and ACR70 scores [72]. A subsequent network meta-analysis demonstrated that decernotinib exhibited the highest efficacy, while filgotinib showed the lowest efficacy, as measured by ACR20, ACR50, and ACR70 response criteria [98]. The lowest mortality incidence was associated with upadacitinib treatment, whereas filgotinib was linked to the highest mortality rate. Additionally, the highest risk of malignancy was observed with baricitinib, while upadacitinib was associated with the lowest malignancy risk [98]. Although these network meta-analyses provide comparative data on the efficacy of JAKi in RA, there is a lack of evidence regarding their relative effects on pain outcomes in RA patients.

## 6. JAK Inhibitors vs. Other RA Treatments for Pain

JAKi are fundamentally different from csDMARDs and biologic therapies in their mechanism of action and breadth of cytokine modulation, which has important implications for pain management in RA. While traditional therapies often aim to suppress inflammation at the systemic level, JAKi act intracellularly to block the JAK-STAT pathway, thereby reducing the expression of multiple pro-inflammatory and pain-associated cytokines, including IL-6, IFN-γ, GM-CSF, and others [99]. This unique pharmacological profile may underlie their more pronounced and rapid analgesic effects.

### 6.1. Comparing JAK Inhibitors with csDMARDs

Methotrexate remains the anchor drug in RA therapy, providing modest pain relief primarily through anti-inflammatory effects [100]. However, its slow onset of action (typically 6–12 weeks), gastrointestinal side effects, hepatotoxicity, and variability in clinical response limit its efficacy in patients with high pain burden [101]. Studies suggest that while MTX may reduce joint swelling and inflammation, its effect on patient-reported pain is often less impressive [100]. In contrast, JAKi, such as tofacitinib and upadacitinib, have demonstrated significant reductions in pain VAS scores within the first 1–2 weeks of initiation, highlighting a critical advantage in pain-dominant disease phenotypes [102].

For example, the ORAL Strategy trial showed that tofacitinib in combination with MTX produced greater improvements in pain scores compared to methotrexate monotherapy [103]. Similarly, in the monotherapy arms of the SELECT-MONOTHERAPY trial, upadacitinib outperformed MTX in both early-onset and sustained pain reduction over 24 weeks [104]. These findings support the preferred use of JAKi in patients with inadequate pain response to csDMARDs or in those who require rapid symptom relief.

### 6.2. Biologic DMARDs and Their Limitations in Pain Control

Biologic agents such as TNF-α inhibitors (e.g., ADA, etanercept), IL-6R inhibitors (e.g., tocilizumab), and T-cell co-stimulation modulators (e.g., abatacept) have revolutionized the treatment of RA [105]. However, their analgesic efficacy varies depending on the cytokine targeted. TNF-α inhibitors have demonstrated substantial efficacy in controlling joint inflammation and structural damage, but their effect on pain, particularly central pain, has been inconsistent [106]. A subset of patients experience persistent pain despite achieving clinical remission by DAS28 criteria, reflecting non-inflammatory mechanisms of pain.

IL-6 plays a central role in neuroimmune crosstalk and central sensitization [107]. As a result, IL-6 inhibitors such as tocilizumab may have superior analgesic properties compared to TNF-α inhibitors. In the AMBITION and ADACTA trials, tocilizumab showed greater improvements in pain and PtGA than MTX and ADA, respectively [108,109]. However, even IL-6 blockade may not be sufficient in some patients with refractory or non-inflammatory pain.

JAKi may overcome these limitations by targeting multiple cytokines simultaneously. This broader scope of immunomodulation not only improves inflammatory control but also provides a better opportunity to attenuate pain pathways. For example, the JAK1 selective inhibitor filgotinib has been associated with reduced pain sensitivity and interference with daily function as assessed by the SF-36 physical pain domain [110]. JAKi provide rapid, robust, and potentially multifaceted pain relief in RA, setting them apart from csDMARDs and even some biologics [111]. Their unique mechanism of action allows them to target both inflammatory and non-inflammatory components of pain [112]. For patients with persistent pain, early aggressive treatment with JAKi may prevent the transition to chronic pain states and improve long-term outcomes compared to conventional treatments.

### 6.3. Clinical Trials Comparing JAK Inhibitors to TNF-α Inhibitors

Numerous pivotal trials have evaluated the analgesic effects of JAKi compared with existing biologics. The SELECT-COMPARE trial compared upadacitinib (15 mg once daily) with ADA (40 mg every other week), both on a background of MTX [113]. Upadacitinib not only demonstrated non-inferiority but also outperformed ADA in pain reduction as early as week 2, with statistically significant improvements in patient-reported VAS sustained through week 48. Similarly, in the RA-BEAM study, baricitinib (4 mg daily) outperformed ADA in improving PtGA and pain scores by week 12 [16].

The superiority of JAKi is further demonstrated in studies such as FINCH 1 (filgotinib vs. ADA), where filgotinib produced greater and more rapid reductions in pain [114]. These results demonstrate not only efficacy in disease control but also meaningful differences in analgesic outcomes, which are of paramount importance to patients.

### 6.4. Comparative Pain Outcomes with JAK Inhibitors Versus Abatacept

Head-to-head comparisons of JAKi are limited but growing. The SELECT-CHOICE trial compared upadacitinib with abatacept in patients with an inadequate response to biologics and showed greater pain improvement with upadacitinib at week 12 [114]. Meanwhile, indirect comparisons suggest minimal differences in pain outcomes between tofacitinib, baricitinib, and upadacitinib, although upadacitinib may have a slightly faster onset and greater magnitude of effect [115].

### 6.5. Network Meta-Analyses: Synthesizing Indirect Evidence

Network meta-analyses have been instrumental in comparing multiple RA therapies in the absence of direct head-to-head trials. A recent network meta-analysis published in 2021 by Weng et al. integrated data from over 45 RCTs and found that JAKi, particularly upadacitinib and baricitinib, consistently outperformed TNF-α inhibitors, IL-6 inhibitors, and csDMARDs in improving pain VAS scores [116]. This supports the hypothesis that the broader cytokine inhibition and intracellular targeting of JAK inhibitors may translate into more robust analgesic effects.

In these network meta-analyses, improvements in pain were often dissociated from reductions in CRP or joint swelling, suggesting mechanisms beyond inflammation control [116]. Importantly, JAKi showed consistency across different patient populations, including those with long disease duration, multiple prior treatment failures, or comorbid fibromyalgia.

### 6.6. Real-World Evidence Supporting Rapid and Sustained Pain Relief

Observational studies and registry data have validated clinical trial findings in real-world settings. Data from the Corrona RA Registry and the OPAL cohort in Australia established that patients treated with JAKi report earlier and greater reductions in pain compared to TNF-α inhibitors or IL-6 inhibitors [117,118]. In the Corrona registry, approximately 40% of patients on JAKi achieved a minimal clinically important difference in pain within 4 weeks, compared to 26% on TNF-α inhibitors [118].

In addition, a 12-month retrospective analysis showed sustained pain relief, with significantly higher patient adherence and satisfaction scores in patients treated with JAKi [119]. Such findings support their utility not only in RCT populations but also in the broader, more heterogeneous patient populations seen in everyday practice.

### 6.7. Special Populations: Comorbid Fibromyalgia and Non-Inflammatory Pain

A particularly challenging subset of RA patients are those with overlapping fibromyalgia or primarily non-disabling pain [120]. These patients often have high pain scores, poor QoL, and suboptimal responses to traditional anti-inflammatory therapies [120]. In such cases, the broad cytokine inhibition and potential central effects of JAKi may offer unique advantages.

Subgroup analyses from SELECT-NEXT and ORAL-Scan suggest that patients with high pain catastrophizing or low correlation between inflammation and pain levels respond favorably to JAKi [121,122]. This raises the possibility that JAKi may be selectively beneficial in discordant pain phenotypes, although more targeted research is needed.

### 6.8. Safety-Tolerability Balance in the Context of Pain Management

The decision to use JAKi for improved pain control must be balanced against their safety profile. The increased risk of herpes zoster, venous thromboembolism, major adverse cardiovascular events, and malignancy has been highlighted by regulatory agencies [123]. However, the absolute risk remains low in appropriately selected patients, particularly younger individuals without major cardiovascular risk factors.

When weighing treatment options, the morbidity associated with persistent, uncontrolled pain must be considered. Chronic pain contributes to physical disability, psychological distress, reduced adherence to treatment, and increased healthcare costs [124]. Therefore, for patients with debilitating pain, the benefits of JAKi in improving function and QoL may outweigh the risks, provided that individualized risk assessment and monitoring protocols are implemented.

## 7. Unmet Needs and Future Directions

### 7.1. Why Some Patients Fail to Achieve Pain Relief Despite JAK Inhibition

RA pain is caused by inflammation, central and peripheral pain processing, and structural changes in the joint [96]. Pain can occur in non-inflammatory areas and may be resistant when RA is in remission [6,125]. Patients with RA also deal with a number of comorbidities, including depression and fatigue. Persistent pain and depression in the absence of inflammation suggest that changes in the CNS caused by inflammatory episodes continue over time, resulting in psychological abnormalities [126].

JAKi reduced pain considerably more than bDMARDs according to the systematic review and meta-analysis of several clinical trials [14,102,127]. This implies that JAKi may have analgesic effects in addition to their anti-inflammatory ones [102]. Numerous investigations have pointed out that no inflammatory pain, most likely due to central sensitization, is the reason why RA patients continue to experience pain [128,129]. The degree of pain that patients experience is unrelated to quantitative assessments of joint inflammation [130,131,132]. Despite major therapeutic advances that have decreased synovitis and disease activity, pain remains a major issue in RA [133].

### 7.2. Predictive Biomarkers for Pain Relief Response

Studies to identify which biomarker, in contrast to traditional activity markers, more accurately depicts disease activity in patients receiving JAKi treatment are not yet available [134]. However, throughout time, precision medicine has discovered several soluble biomarkers that are predictive of diagnosis, prognosis, and therapy response in RA, including myeloid-related protein 8/14, cellular, and autoantibodies (ACPA, anti-carbamylated protein antibodies 14-3-3, and anti-protein-arginine deiminases antibodies 3/4) [135]. The pathogenetic onset and progression of cartilage damage in RA have been strongly associated with the urokinase plasminogen activator (uPA) protease in recent years [130]. It has been discovered that serum levels of soluble uPA receptors reflect joint degeneration over time and correlate with disease activity in early RA [136]. The relationship between circulating calprotectin and RA has also been investigated [137].

### 7.3. Emerging JAK Inhibitors and Novel Therapeutic Targets in RA Pain

JAKi are divided into two categories based on their selectivity: first-generation JAKi, which include non-selective inhibitors, and second-generation, which block the signaling of a more limited set of cytokines [138]. First-generation JAKi encompass baricitinib and tofacitinib; the second-generation JAKi include upadacitinib, decernotinib, filgotinib, peficitinib, and itacitinib; some are still under development. When taken as monotherapy, second-generation JAKi appear to be more effective, more rapid, and dose-dependent in comparison with first-generation [139].

Upadacitinib, a selective JAK1 inhibitor, is the third JAKi authorized for RA. Its effectiveness in treating RA has been proven by several clinical investigations [81,104]. Numerous clinical studies have shown a statistically significant improvement in ACR response rate as well as pain and fatigue rated by patients [81,104].

Peficitinib is an oral JAKi approved in 2019 in Japan for the treatment of RA [140]. Compared to other second-generation JAKi, peficitinib exhibits lower target selectivity [139]. It is a pan-JAK inhibitor, like tofacitinib, having a greater selectivity for JAK3 than for the other JAKi [141]. The studies showed a statistically significant improvement in the ACR20 and ACR 50 response rate at weeks 12 and 52 and a similar number of adverse events compared with placebo [142].

Filgotinib potently and selectively inhibits JAK1 [140]. Compared to unselective JAKi, filgotinib is thought to have a better safety profile and good efficacy because of its target selectivity [138]. Numerous clinical studies have demonstrated its efficacy in treating RA [84,143,144]. During the observational period, no opportunistic infections, malignancies, or deaths were noted [138].

Decernotinib is an oral selective JAK3 inhibitor whose development for the treatment of RA is presumed to have been discontinued [138].

Itacitinib is a new oral selective JAK1 inhibitor that was authorized by the EMAAVCI in 2018 as an orphan medication to treat graft versus host disease [145]. However, no registration trials have been planned for itacitinib’s usage in RA as of yet [138]. A summary of emerging JAK inhibitors is given in Table 5.

### 7.4. Personalized Pain Management Approaches in RA

The complex interplay between central sensitization, peripheral inflammation, and psychological factors underscores the need for an individualized and multidimensional approach to pain management and assessment in patients with RA [146]. A thorough evaluation of the origins, mechanisms, and functional consequences of pain in each individual is critical, as pain in RA may result not only from inflammatory activity but also from structural joint damage, altered pain processing, or concomitant comorbidities. Accordingly, management strategies should integrate pharmacological, psychosocial, and physiotherapeutic interventions tailored to the specific needs of each patient [96].

Understanding comorbid conditions, polypharmacy, and the broader psychosocial context is crucial in determining the optimal risk–benefit balance of therapeutic options [96]. Patients should be supported in making informed decisions regarding analgesic use, recognizing that individual preferences and risk perceptions often vary considerably [96,147]. Beyond conventional analgesics, treatment strategies for RA pain may encompass immunomodulatory drugs, cognitive-behavioral and other psychosocial therapies, structured exercise and physical activity programs, and, in selected cases, surgical interventions [133].

Patient stratification using biomarkers, neuroimaging, and patient-reported outcomes to identify unique pain profiles and adjust treatment is becoming more and more important in emerging methods for individualized pain management. While patients with predominantly inflammatory pain may benefit more from aggressive disease-modifying medication, clinicians may be guided toward interventions that target neural pain pathways if they identify those with a predominance of central sensitization. Furthermore, wearable technology, telemedicine platforms, and digital health tools present new possibilities for joint decision-making, ongoing pain monitoring, and dynamic therapy modification. In the end, a comprehensive and multidisciplinary approach that incorporates the biological, psychological, and social facets of health is necessary for tailored pain treatment in RA in order to enhance quality of life and symptom control.

### 7.5. Future Directions

Even with significant advancements, there are still significant unanswered questions about how best to utilize JAKi to treat RA pain. Finding accurate biomarkers that predict individual response should be the top priority of future research in order to enable a precision medicine approach to drug selection. Optimizing medication allocation may be possible by stratifying patients based on the predominant pain mechanisms, such as centrally mediated, nociceptive, or inflammatory, through the integration of genomic, transcriptomic, and proteomic profiling.

The development of novel therapeutic strategies remains essential due to the limitations of current RA treatments. Targeted suppression of IL-2 expression represents a promising approach to modulate pathogenic immune responses [148]. Subcutaneous MTX offers advantages over oral administration, including reduced gastrointestinal side effects and improved disease control. Emerging research is also exploring innovative biologics and nanotechnology-based therapies, such as cell membrane-coated nanoparticles, which enable targeted drug delivery with enhanced anti-inflammatory efficacy, improved safety, and potentially reduced dosing frequency [149].

The permanence of pain relief and the relative safety of JAKi, especially in relation to cardiovascular, infectious, and malignancy concerns in a variety of patient populations, require long-term observational studies and empirical data. In order to achieve synergistic effects on pain and overall disease burden, parallel efforts should investigate the potential role of JAKi in combination strategies, either with biologics, conventional DMARDs, or non-pharmacological modalities like structured exercise or cognitive-behavioral therapy.

Digital health innovations and remote monitoring technologies have the potential to further improve care personalization by recording patient-reported outcomes and pain variations in real time, which may then be used to influence dynamic therapy modifications. Ultimately, mechanistic research that connects neuroimmune modulation to clinical outcomes will be essential to improving our knowledge of how JAK inhibition reduces chronic pain and possibly expanding therapeutic insights to other pain disorders, both rheumatic and non-rheumatic.

When considered collectively, these potential paths forward demonstrate the potential of JAKi as both strong anti-inflammatory drugs and essential elements of a comprehensive, mechanism-based strategy for individualized pain management in RA.

## 8. Conclusions

Pain in RA remains a major unmet need, often persisting despite inflammatory control. The JAK-STAT pathway not only drives synovial inflammation but also contributes to central sensitization, neuroinflammation, and chronic pain. JAKi, by modulating multiple cytokines implicated in both peripheral and central pain processing, has redefined pain management strategies in RA.

Beyond suppressing inflammation, JAKi demonstrate direct analgesic effects, improving patient-reported pain, fatigue, and physical function. Clinical trials and real-world data (although still scarce) consistently point towards the aforementioned, more robust pain relief with JAK inhibition compared to conventional synthetic DMARDs and some biologic agents. However, variability in patient response, persistence of nociplastic pain, and safety considerations underscore the need for precision medicine approaches.

Future research must focus on elucidating the mechanisms by which JAKi modulate central pain networks, identifying biomarkers predictive of analgesic response, and optimizing personalized treatment strategies. Integrating JAKi into comprehensive pain management paradigms offers a promising pathway to improving QoL for RA patients. Understanding pain as a multifaceted phenomenon—beyond mere inflammation—is critical to advancing therapeutic outcomes in RA.

## Figures and Tables

**Table 1:** A summary of currently available JAK inhibitors.

JAKi	Selectivity	Recommended Dose	FDA Approved	EMA Approved
tofacitinib	JAK 1 and JAK 3 (strong) JAK 2 (minor) TYK 2 (minor)	2 × 5 mg	11/2012	3/2017
baricitinib	JAK 1 and JAK 2 (strong) TYK 2 (moderate) JAK 3 (minor)	2 mg 4 mg	5/2018	12/2016
upadacitinib	JAK 1 (strong)	15 mg	8/2019	12/2019
filgotinib	JAK 1 (strong)	100 mg 200 mg	-	9/2020

Abbreviations: EMA—European Medicines Agency FDA—Food and Drug Administration; JAKi—Janus kinase inhibitors.

**Table 2:** Key mechanisms in JAK-STAT signaling in RA [39,40,41].

Mechanism	Activated Pathway	Cells/Tissues Involved	Pathophysiological Outcome	Clinical Phenotype/ Relevance	Therapeutic Implication
IL-6/JAK/STAT3 Axis	IL-6 → JAK1/JAK2/TYK2 → STAT3	Synovial cells, T cells	Inflammation, osteoclast activation, angiogenesis	Inflammatory pain, active disease phase	Key therapeutic target, biomarker of disease activity
Predominant Role of JAK1	IL-6, IFN → JAK1 → STAT1/3	Synovial fibroblasts, osteoclasts	Synovial inflammation, bone remodeling	Erosive disease, disease progression	Selective JAK1 inhibition as a targeted strategy
Impaired JAK/STAT Activity	Cytokines ↑, but ↓ STAT phosphorylation	PBMCs, T cells	Discordant inflammation-signaling response	Poor therapeutic response, disease heterogeneity	Basis for personalized therapeutic approaches
Role of SOCS Proteins	SOCS2 ↓ → loss of JAK/STAT regulation	T cells, macrophages	Loss of negative feedback, chronic inflammation	Persistent disease, chronic pain	Potential target for SOCS modulation therapies

**Table 3:** Summary of JAK inhibitors’ mechanism affecting pain pathways.

Authors	JAK Inhibitors	Type of Study/Methods	Mechanism Affecting Pain Pathway
Makabe et al. [50]	baricitinib	CAIA mice model	- Suppression of arthritis-dependent pain - Suppression of tactile allodynia, a critical feature of neuropathic pain - Downregulation of the inflammatory response pathways and Csf1 gene expression (cytokine related to neuropathic pain) in DRG - Suppression of the neuropathic pain-related changes, such as microglial and astrocyte proliferation in the SDH - Suppression of the IL-6 induced expression of Csf-1 in cultured neuronal cells
Vazquez et al. [52]	baricitinib	C57BL/6J mice and Wistar rats, primary cultures of DRG neurons	- Pain attenuation through direct effects on sensory neurons - Suppression of the sensitizing effect of IL-6 + sIL-6R on nociceptive C-fibers (reduced response to mechanical stimulation) - Prevention of IL-6 + sIL-6R induced neuronal hyperexcitability in DRG neurons in vitro
Simon et al. [53]	baricitinib	CAIA mice model, DRG neurons culture	- Reversal of mechanical hypersensitivity and pain-related behavior - Suppression of synovial hyperinnervation and STAT3 phosphorylation in DRGs - Inhibition of AAK1-mediated signaling, which is related to mechanical hypersensitivity - Decrease in neuronal excitability in DRG cultures
Matsushita et al. [55]	baricitinib	CIA mice model	- Suppression of microglial activity in the central nervous system—area postrema region - Improvement in pain-related behavior
Navarini et al. [64]	upadacitinib	Microglia culture	- Decreasing the level and production of BDNF (molecule involved in modulation of nociception and pain signaling) in IL-6 treated microglia
Li et al. [65]	abrocitinib	TBI mice model, BV2 microglial cell culture	- Promoting the polarization of microglia toward the anti-inflammatory phenotype M2 while reducing the pro-inflammatory M1 pool
Tsuda et al. [57]	AG490	Neuropathic pain model—male Wistar rats	- Suppression of proliferation of dorsal horn astrocytes and relieved tactile allodynia
Tian et al. [59]	AG490	Electrophysiological recordings- Sprague Dawley rats and C57BL/6J mice	- Attenuation of leptin-induced NMDA currents in dorsal horn neurons - Possible role in alleviation of thermal hyperalgesia and mechanical allodynia

Abbreviations: JAK: Janus kinase, CAIA: collagen antibody-induced arthritis, CIA: collagen induced arthritis, CSF: colony-stimulating factor, DRG: dorsal root ganglion, SDH: spinal dorsal horn, IL-6: interleukin-6, sIL-6R: soluble interleukin-6 receptor, STAT3: signal transducer and activator of transcription 3, BDNF: brain-derived neurotrophic factor, NMDA: N-methyl-D-aspartate.

**Table 5:** Summary of emerging JAK inhibitors.

Emerging JAKi	Approved	Indication	Selectivity
Peficitinib	Japan, 2019	RA	JAK 3 (strong) JAK 1 and JAK 2 (minor) TYK 2 (minor)
Decernotinib	-	RA	JAK 3 (strong)
Itacitinib	-	RA	JAK 1 (strong)

Abbreviations: JAKi—Janus kinase inhibitors; RA—rheumatoid arthritis.

## Data Availability

The data underlying this article will be shared on reasonable request to the corresponding author.
